# Artificial Stone Associated Silicosis: A Systematic Review

**DOI:** 10.3390/ijerph16040568

**Published:** 2019-02-16

**Authors:** Veruscka Leso, Luca Fontana, Rosaria Romano, Paola Gervetti, Ivo Iavicoli

**Affiliations:** 1 Section of Occupational Medicine, Department of Public Health, University of Naples Federico II, Via S. Pansini 5, 80131 Naples, Italy; veruscka.leso@unina.it (V.L.); rosaria_romano@libero.it (R.R.); gervettipaola@gmail.com (P.G.); 2 Department of Occupational and Environmental Medicine, Epidemiology and Hygiene, Italian Workers’ Compensation Authority (INAIL), Via di Fontana Candida 1, Monte Porzio Catone, 00040 Rome, Italy; lfontana73@yahoo.it

**Keywords:** artificial stone, engineered stone, reconstituted stone, artificial quartz, silicosis, occupational exposure, exposure evaluation, risk assessment, risk management

## Abstract

Silicosis is a progressive fibrotic lung disease that is caused by the inhalation of respirable crystalline silica. Due to its high silica content, artificial stone (AS) can become a possible source of hazardous dust exposure for workers that are employed in the manufacturing, finishing, and installing of AS countertops. Therefore, the aim of this review was to verify the association between AS derived silica exposure and silicosis development, and also then define the pathological characteristics of the disease in relation to specific work practices and preventive and protective measures that were adopted in the workplace. A systematic review of articles available on Pubmed, Scopus, and Isi Web of Knowledge databases was performed. Although the characteristics of AS-associated silicosis were comparable to those that were reported for the disease in traditional silica exposure settings, some critical issues emerged concerning the general lack of suitable strategies for assessing/managing silica risks in these innovative occupational fields. Further research that is designed to assess the hazardous properties of AS dusts, levels of exposure in workplaces, and the effectiveness of protective equipment appears to be needed to increase awareness concerning AS risks and induce employers, employees, and all factory figures that are engaged in prevention to take action to define/adopt proper measures to protect the health of exposed workers.

## 1. Introduction

Silicosis is a progressive, irreversible, and incurable fibrotic pulmonary disease that is caused by the inhalation of respirable crystalline silica (RCS) dust [1]. Key factors determining the risk of developing silicosis include lifetime cumulative exposure, total amount of inhaled RCS, and individual susceptibility [2,3]. Mechanistically, when respirable silica particles are inhaled, they can reach the lower respiratory tract and the gaseous exchange zones where, after having been phagocytosed by alveolar macrophages, they can persist and then trigger an inflammatory process that is characterized by the production of reactive oxygen species (ROS) [4,5]. The inflammation that is generated by ROS damages the pulmonary parenchyma and the subsequent repair/regeneration process leads to fibrogenesis and carcinogenesis [1,4,5]. Respirable dust control represents the only effective measure to prevent disease manifestation and no curative therapies are currently available [6].

Crystalline silica is a common component of the earth’s crust and it can be found in quartz, granite, sandstone, slate, and sand [7,8]. It is widely acknowledged that occupational exposure to crystalline silica may occur in several workplaces and industries, such as the construction and metallurgy industries, coal and metal mining/quarrying, and the manufacturing of building materials (e.g., bricks and concrete), glass, and ceramics [7,8]. Therefore, when considering the large number of industrial applications and working activities that involve the use or handling of materials containing silica, it is estimated that millions of workers are exposed to this mineral worldwide (approximately 10 million in India, 3.2 million in the European Union, 2.3 million in the United States of America (USA), and 2  million in Brazil) [9,10,11,12]. Therefore, silicosis is a major work-related interstitial lung disease [1,13,14,15,16]. More recently, the manufacturing and processing of artificial stone (AS) has been reported as a possible source of exposure to high levels of RCS in workers [17].

In recent decades, this specific type of material has become increasingly popular and it has been largely employed for the production and manufacturing of kitchen and bathroom countertops. Artificial stone is formed of finely crushed rocks that are mixed with a polymeric resin. Its silica content is approximately 90%, a much higher percentage than the silica content of natural marble (3%) or granite stones (30%) [18]. Through the cutting and grinding of AS slabs with high-energy, powerful devices may result in high levels of exposure to RCS dusts, although little information is currently available regarding concentrations in these specific workplace settings/tasks [19,20,21,22]. It should be noted that the Occupational Safety and Health Administration and the National Institute for Occupational Safety and Health have identified exposure to silica as a “health hazard to workers involved in manufacturing, finishing and installing natural and manufactured stone countertop products, both in fabrication shops and during in-home finishing/installation” [23].

Indeed, the concern that is aroused by this hazard alert has been further corroborated by the findings of several studies that have highlighted outbreaks of silicosis among AS workers in various countries throughout the world [24,25,26,27,28,29,30,31,32,33,34,35,36,37]. Moreover, it is worth pointing out that the growing concern of the scientific community with regard to this topic is related, not only to an increased incidence of the disease in AS workers, but also to the different pathological characteristics and the high degree of severity of AS-associated silicosis. In fact, most epidemiological or clinical studies reported cases of accelerated silicosis characterized by a short latency period, extensive pulmonary damage, and its presence in young workers. The greater aggressiveness of AS-associated silicosis is usually attributed to a lack of adequate preventive or protective measures: this may be a plausible explanation given the high levels of exposure that is generated over a short period of time. However, a recent interesting in vitro study of Pavan et al. [38] showed that AS dusts exhibited a higher reactivity in free radical production when compared to reference quartz. The authors correlated this result to the larger amount of metal transition ions that were contained in the AS dusts, therefore also suggesting that the different chemical features could play an important role in the pathogenesis of AS-associated silicosis [30,38].

Therefore, by means of a systematic and critical analysis of the available literature, the aim of this review was to verify the association between RCS exposure in AS working activities and the development of pulmonary silicosis; to define the common pathological characteristics that could promote the onset of this disease in relation to specific job tasks or work practices, and also in highlighting research areas that require further investigation. Overall, this may be important in extrapolating data that provide useful information on the risk of silicosis in AS production/working fields and then subsequently lead to suitable risk assessment and management strategies to adequately protect the health of exposed workers.

## 2. Materials and Methods

**Figure 1:**
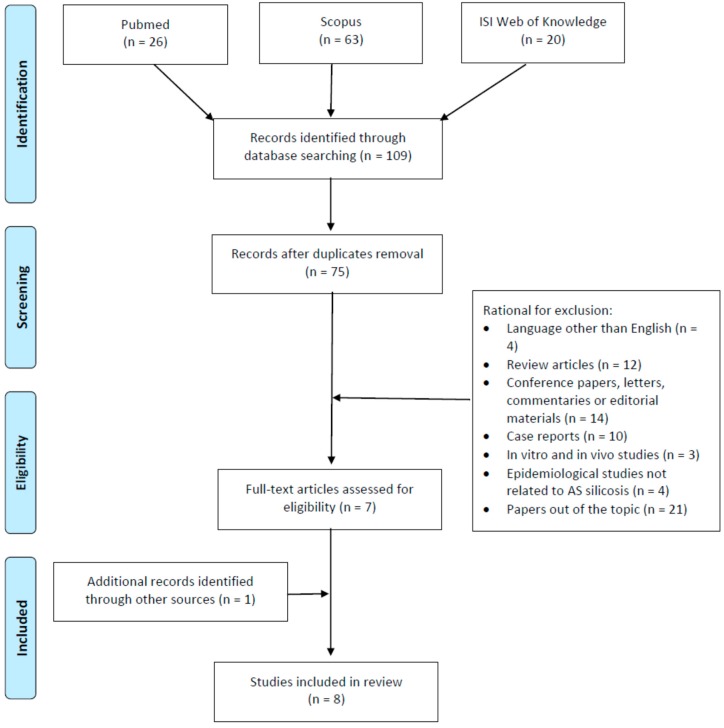
Flow diagram of literature search.

The study involved a systematic review process that was performed according to the Preferred Reporting Items for Systematic Reviews and Meta-Analyses Statement criteria (PRISMA) [39]. Principal scientific databases, namely PubMed, Scopus, and ISI Web of Science were searched to identify studies addressing cases of AS-associated silicosis, published up to 15 December 2018. We used two search lines that included the terms “artificial stone or artificial quartz or engineered stone or reconstituted stone” to assess the exposure context, and the term “silicosis” to identify the outcome. The two lines were combined with the operator ”AND”. All of the titles and abstracts that were retrieved by the computerized search were independently reviewed by two of the authors who made a selection of the papers that are relevant for the review purposes, in accordance with the inclusion criteria. These referred to original, human peer-reviewed articles, including descriptive epidemiological-occupational surveys, medical reports, case series, cohort and case-control studies published in English, and reporting cases of silicosis and AS exposure. To be included in the review, studies had to describe pathological cases that were confirmed through valid diagnostic methods, including clinical examination, pulmonary function tests, and imaging techniques. Occupational exposure to AS dust had to be confirmed by occupational histories of employment in any sector of the manufacturing, finishing, and installation of AS, as collected through medical records, patients’ self-reported information, as well as through environmental monitoring when available. No limits regarding the duration of AS occupational exposure were adopted, and no restrictions were imposed on the geographical areas of investigation, patient origin, or the statistical methods used. Exclusion criteria regarded reviews, case reports, conference papers, experimental studies on cellular and animal models, and publications that did not focus on occupational exposure to AS dust or that were were published in languages other than English. The preliminary search identified a total of 109 articles: 26, 63, and 20 in Pubmed, Scopus, and Isi Web of Knowledge databases, respectively. Thirty-four duplicates were removed from the total number of papers. Out of the remaining 75 articles, two authors independently excluded 68, as they did not meet the inclusion criteria based on the title and abstract analyses. A total of seven papers remained for review. All of the full texts of the articles that were considered suitable for review were obtained and subjected to a critical evaluation. By assessing the reference list accompanying the selected articles further enlarged the citation pool of relevant publications that were identified in the literature search; this allowed for the inclusion of one additional eligible paper. Overall, our search retrieved a total of eight articles for review (Figure 1).

Each eligible study was critically reviewed by three investigators and the principal characteristics were extracted in order to determine the demographic and occupational characteristics of cases, disease features, and workplace information. The Joanna Briggs Institute (JBI) Critical Appraisal tools for use in JBI Systematic Reviews Checklist for Case Series was used to assess the methodological quality of a study and to determine the extent to which a study has addressed the possibility of bias in its design, conduct, and analysis [40]. 

The results of the eligible studies are described in the following sections and then organized into tables summarizing information concerning case identification, periods of investigations, geographical areas of origin, mean or median age of affected subjects, working activities in which AS exposure could occur, and the duration of exposure where available, as well as quality rating, as assessed through the JBI checklist (Table 1). To define pathological manifestations of AS-associated silicosis, data concerning clinical examination, respiratory function tests, as well as radiological findings were collected and are summarized in Table 2. Information on histological findings and disease outcome following lung transplantation was also reviewed to complete the overview of the pathology. Furthermore, to extrapolate information regarding risk assessment and management strategies for dealing with AS chemical risks in workplaces, data concerning exposure assessment, as well as preventive and protective measures that were adopted to protect the health and safety of exposed workers, were carefully collected, evaluated, and reported, as shown in Table 3.

## 3. Results

The following paragraphs will summarize details regarding cases of AS-associated silicosis that are emerging worldwide, and the principal characteristics of the disease.

### 3.1. Identification of Cases

In recent years, a silicosis outbreak that was attributed to occupational AS exposure was reported in Israel [7]. This was the first, large, retrospective series of 25 AS silicosis cases occurring in patients that were admitted to the National Lung Transplantation Center in Israel to be evaluated for lung transplantation (LTX) in the period 1997–2010 [7]. According to their occupational histories, all of the patients had been working with the same synthetic stone material for a period ranging from 17 to 22 years and they were all mainly involved in AS dry cutting for end-use countertop application. An updated evaluation of patients referred to the same center for advanced silica-related pulmonary pathology, over a further two-year period of observation (1997–2012), enabled researchers to identify 15 additional cases of silicosis related to AS dust (a total of 40 cases) in workers that were exposed for at least six years [41]. Eighty-two Israeli marble workers that were affected by AS silicosis were studied by computed tomography (CT) and pulmonary function tests (PFTs) by Grubstein et al. [42] from 1997 to 2015, while Rosengarten et al. [16] retrospectively reviewed the post-lung transplantation condition in 17 silicosis patients from 2006 through 2013. All of the subjects had been working with AS. However, a possible overlap between the above-mentioned populations prevents the extrapolation of a global count of Israeli cases of the disease, therefore limiting an assessment of the real impact of AS silicosis in that country.

A high incidence of silicosis was retrospectively detected over a short period of time (2009–2012) in the small geographical area of the Cadiz Province of Spain [43]. Forty-six workers, which were employed in the manufacture and installation of kitchen bench-tops composed of quartz conglomerates over a period ranging from nine to 17 years, were diagnosed with silicosis. They had generally been involved in AS cutting, shaping, and finishing in small family industries. A prospective, observational study that was performed by Pascual et al. [44] reported six cases of silicosis in a cohort of 11 workers from a family marble workshop in Spain. All except one employee had been working for an average of 12.5 years as countertop assemblers and had been involved in the cutting and polishing of AS during in-home installation. The same authors, in a follow-up study, described a total of 19 cases of silicosis in the region of Valencia from 2009 to 2016. Twelve of these cases occurred in assemblers, cutters, and sanders that are involved in the finishing of kitchen and bath countertops over an average of 11 years [45].

Between 2011 and 2016, seven cases of AS-associated silicosis were detected in Australia: one in Queensland and three in Victoria and New South Wales, respectively [17]. All cases had been employed for a period of 4–10 years in small kitchen and bathroom benchtop fabrication where the dry cutting of AS was one of the most frequently performed tasks (Table 1).

### 3.2. Symptoms, Pulmonary Function Tests, Radiological Diagnosis

Mild respiratory symptoms were reported in 82.6% of the Spanish population that was investigated by Perez Alonso et al. [43], while the remaining 17.4% was asymptomatic. All workers of the Australian series (n. 7) had cough and exertion shortness of breath, while only two suffered from weight loss and haemoptysis [17]. The duration of symptoms before diagnosis ranged from six months to three years. Taking into account the well-known association between silica exposure and autoimmune diseases, Shtraichman et al. [41] identified nine patients, among the 40 that were investigated for lung transplantation, who were co-diagnosed with silicosis and systemic sclerosis (n. 3), Sjogren’s syndrome (n. 1), rheumatoid arthritis (n. 2), polymyosisitis (n. 1), and mixed connective tissue disease (n. 2), respectively. 

The assessment of pulmonary functionality in Israeli patients (potential candidates for lung transplantation) revealed moderate-to severe restrictive lung disease in all cases [7] (Table 2). Similarly, a restrictive pattern and a reduced diffusing capacity for carbon monoxide (DLCO) was evident in all but one of the subjects that were studied by Shtraichman et al. [41] for autoimmune manifestations of AS silicosis. Spanish workers that were affected by simple silicosis (n. 42) showed a moderately restrictive pattern at the PFTs, while the four complicated cases demonstrated a more restrictive spirometric profile and a greater reduction in DLCO [43]. Restrictive functional patterns were also reported in six out of seven Australian silicosis cases [17]. Pascual et al. [44] demonstrated ventilatory disorders with reduced DLCO in three out of six diagnosed workers and, interestingly, one of these showed moderate obstructive function. When PFTs were compared between AS silicosis and idiopathic pulmonary fibrosis transplanted patients in Israel, the first group demonstrated a lower forced expiratory volume in 1 s (FEV1). This may have been due to the possible mixed restrictive and the obstructive pattern that is common in silicosis [1,16,46].

Concerning radiological diagnosis, which is in line with clinical silicosis manifestations, a bilateral diffuse micronodular pattern was detected at chest X-ray in 80.4% (n. 37) of the cases that were investigated by Perez-Alonso et al. [43], while normal findings were evident in 19.6% of the examinations (Table 2). The high resolution-CT revealed simple and complicated chronic silicosis in 91.3% and 8.7% of cases, respectively. A reticulonodular interstitial pattern was evident at the chest X-ray imaging in eight out nine patients with concomitant autoimmune diseases, as reported by Shtraichman et al. [41]. In Hoy et al. [17], CT scans revealed semiconfluent nodules in the mid and upper zones of the lungs, with increased upper lobe interstitial markings (two subjects), extensive ground glass nodules (two patients), as well as a pattern of progressive massive fibrosis that is characterized by large confluent mass-like densities and volume loss (6 subjects). Grubstein et al. demonstrated a significant inverse correlation between the severity of the chest CT findings and the PFT parameters, particularly in regards to the FEV1 and the total lung capacity [42]. These authors showed a progressive massive fibrosis indicating advanced and complicated silicosis in 85% of LTX patients and in 40% of affected subjects with conserved pulmonary function.

### 3.3. Histopathological Examination

Peripheral and centrilobular patchy pulmonary fibrosis, with silicosis nodules containing birefringent particles that are consistent with silica, were the typical histological findings from explanted lungs of patients that were studied in Kramer et al. [7]. In the seven Australian silicosis cases, Hoy et al. [17] described inflammatory infiltrates characterized by sclerotic nodules that are surrounded by histiocytes or histiocytic aggregates, sometimes including silica particles. Histological features of silicosis were also found in patients with concomitant autoimmune manifestations [41], with two out of nine patients presenting focal or widespread silico-proteinosis reactions. In a more severe case, in which the subject underwent lung transplantation, extensive areas of confluent fibrosis and silico-proteinosis-like patterns with the presence of birefringent particles were detected.

### 3.4. Post-Lung Transplantation Outcomes

The only life-saving therapeutic option in end-stage silicosis is LTX [16]. Data that are available on the survival outcomes of LTX receiving patients for silicosis or other diseases are conflicting [47,48], and little information has been reported for AS silicosis patients. 

Concerning the one-year survival rate following LTX, Kramer et al. [7] reported comparable rates between patients with AS silicosis and other LTX recipients referred to the National Lung Transplantation Center in Israel over a 14-year period (1997–2010) (83 ± 4% vs. 81 ± 7%, respectively). In a subsequent investigation, Rosengarten et al. [16] specifically compared the follow-up condition of 17 transplanted AS silicosis subjects with 73 patients who underwent LTX for idiopathic lung fibrosis (IPF) in the same medical center in Israel from 2006 to 2013. Patients with silicosis were significantly younger than the IPF patients (mean age: 49.8 versus 57.1 years). The one- and three-year survival rates were 88% and 76% for silicosis patients, respectively. In IPF patients, those rates were 68 and 64%, respectively. Although it was possible to identify a 32% increase in survival outcomes in silicosis patients, the limited amount of data on LTX in similarly-affected individuals prevented researchers from ascertaining statistical significance for this result.

### 3.5. Silica Exposure Risk Assessment and Management

The following paragraphs will attempt to elucidate specific preventive and protective measures that were adopted in AS workplace settings in order to determine the conditions that may promote the onset of disease and identify aspects that need to be carefully considered, so that precautionary risk assessment and management strategies can be undertaken in this field (Table 3).

#### 3.5.1. Exposure Assessment

No environmental monitoring data were reported in AS workplaces where the cases of silicosis reviewed in this study occurred. Kramer et al. [7] failed to report data quantifying the airborne dust concentrations in the AS workplaces that were implicated in the Israeli outbreak of silicosis and declared that they were not aware of any industrial hygiene measurements that were performed by governmental institutions. Rosengarten et al. [16] reported environmental levels of RCS greater than 1 mg/m^3^ in AS dry cutting operations, as measured by the Israel Ministry of Labor, i.e., concentrations ten-times higher than the environmental exposure limit that was adopted in that country. In the province of Perez, Spain, according to worker responses, periodical measurements of dust levels were never performed in any workplace that was involved in AS activities [43]. Although possible recall bias may affect such responses, the authors indicated that the inspections of the workplaces that were carried out by the Centre for Risk Prevention of the Regional Labour Authority and the Inspectorate of the Ministry of Labour in Cadiz supported the results that were obtained through individual interviews. Also, in the Australian case series, workplace exposure monitoring was reported to be unavailable [17].

#### 3.5.2. Collective Protective Measures

Regarding protective measures applied in AS workshops, all of the patients in Kramer et al. [7] reported that their work activities were performed without dust exposure control, including both wet cutting and local exhaust ventilation. In the Spanish population that was examined by Perez-Alonso et al. [43], water curtains, as a means of reducing dust production in AS bench-top cutting activities, were reported to have been adopted in only 32.6% of the workplaces. Engineering controls for workplace ventilation were described to properly in function in 10.9% of cases, and to be ineffective in 54.3%. In the remaining cases (34.8%), working in an outdoor space or in the presence of windows and doors was described as the only ventilation system in the workplace. All of the workers reported the absence of dust ventilation devices during in-home installation operations. Concerning the maintenance of machinery and tools, the same group of patients referred that these were systematically or occasionally (in case of malfunction) serviced, in 6.5% and 26.1% of cases, respectively. In Pascual et al. [44], the affected workers reported the presence of exhaust ventilation systems in their workplaces, in addition to the presence of doors and windows that are designed to guarantee a passive airflow in the occupational environments. Waterjet cutting systems were only used in workshop operations. Installers, which are involved in cutting and polishing AS pieces during in-home installation, were not provided with this kind of prevention system. Likewise, inadequate ventilation and collective protective measures were reported by the same authors in a subsequent investigation [45].

All of the workers in the case series of Hoy et al. [17] performed AS dry cutting, mainly using hand devices, while water dust control was more frequently applied during polishing tasks or when table saws were used. The presence of doors and windows, together with ceiling extraction fans, ensured ventilation in the workplaces.

#### 3.5.3. Personal Protective Equipment

As regards personal protective equipment, workers that were investigated by Kramer et al. [7] reported working without any personal respiratory protection for an average of 10 to 12 h daily. Only 32.6% of the cases that were described by Perez Alonso et al. [43] reported using complete personal protective equipment, including a mask, goggles, gloves, special footwear, and a helmet. Regarding higher protection masks, half of the workers declared that they had had access to such equipment for only a part of their working time, and an even lower percentage (6.5%) reported that this equipment was constantly available. In the study carried out by Pascual et al. [44], home installation of pieces was described as performed without any personal protection. In the clinical series of seven Australian workers [17], respiratory protection was provided for only three subjects. 

#### 3.5.4. Occupational Health Surveillance

Health surveillance programs in the study by Perez Alonso et al. [43] showed some discrepancies that were related to the fact that, during periodic medical examinations, in 32.6% of cases, chest X-ray was never performed, in 58.7%, it was performed only once at the beginning of the observational period, and in a more limited 8.7%, it was obtained periodically. Hoy et al. [17] reported that no organized health surveillance program was carried out for any of the investigated cases. 

## 4. Discussion

This review represents an attempt to provide an updated overview of the current state of knowledge regarding silicosis in the AS manufacturing field. Its aim is to enhance the awareness of the well-known silica health hazard in new occupational exposure realities, and extrapolate data that may be of use in indicating more suitable risk assessment and management strategies in these environments.

Although most of the reviewed studies are observational in nature, therefore impeding a definite association between occupational exposure to RCS in AS activities and silicosis development, the unusually high incidence of the disease that was reported over short periods of investigations, and the comparable occupational histories of affected workers, all being involved in the manufacture and manipulation of engineered stones, may indicate a cause-effect relationship of this type.

In general, the characteristics of AS-associated silicosis, in terms of clinical and latency periods of manifestation, pulmonary functionality alterations, and radiological outcomes, were comparable to those that were reported for the disease in traditional silica exposure settings [17]. In some cases, shorter latency periods, i.e., 4–10 years, were reported before disease development, which may be in relation to the higher intensity of exposure that may characterize some specific job tasks in this field, e.g., cutting, polishing, and grinding AS in workshops and during the in-home installation of pieces, which may generate high levels of RCS [7,43]. Moreover, further clarification is needed as to whether newly fractured silica that are produced by high-energy cutting and abrasive blasting operations, such as those performed by assemblers, cutters, and sanders of countertops, is more toxic than aged powder containing silica in inducing fibrogenic effects due to the greater redox potential on crystal surface [21,22,38]. Additional research is also needed to define the hazardous properties of AS dusts on account of their possible specific toxicological properties resulting from the mixed composition of crushed rock and polymeric resins [38]. The possibility that different components may increase the toxicity of the dust and therefore change the occupational risk profile for workers that are employed in this sector should be carefully considered [30,38].

The main limitations of the studies reviewed are due to the lack of data on environmental monitoring measurements to quantify CRS exposure levels. A couple of case reports, in the finishing areas of artificial quartz manufacture, revealed the average crystalline silica airborne concentrations ranging from 0.260 to 0.744 mg/m^3^ [22] and >0.5 mg/m^3^ [32], which are much higher than the 0.1 mg/m^3^ threshold limit value that was recently adopted in the European Union [49]. When the efficacy of dust control measures in cutting operations was assessed, the dry activities were found to generate a RCS concentration of 44 mg/m^3^ over 30 minutes of sampling. This level decreased to 4.9 mg/m^3^ through the employment of wet blade cutting and it was further reduced to 0.6 mg/m^3^ when the latter measure was combined with local exhaust ventilation [20]. In the studies reviewed, general information on exposure and the preventive measures that were adopted for its control were largely based on the statements of patients and employers, and may therefore have been affected by a recall bias of respondents [43]. The importance of assessing worker exposures depends on the possibility of obtaining information that strongly correlates dust contact and pulmonary effects in the AS production sector. This may also be helpful in defining the possible influencing variables that are related to specific job tasks and work practices that may affect RCS concentrations, and therefore present risks for employees, including, for example, cutting without waterjet machines, as well as polishing without prevention during in-home finishing/installation operations. 

Most of the studies reviewed reported that basic preventive measures for controlling occupational exposure and for protecting the health of workers were not adopted, or not properly adopted. In fact, no effective measures, such as general or mounted-tool local exhaust ventilation systems or wet-cut methods, were implemented to suppress dust generation/exposure when working on AS [20,50,51]. In addition, machinery and tools were not properly set up and they did not undergo the prescribed routine checks [43]. This seems to be a relevant problem, especially for smaller companies that are less aware of occupational safety and health resources than larger factories [12]. Proper compliance with personal protective measures is very important. Most workers reported that full protective equipment was not available, it was not used in the proper manner, and that they did not have access to masks that are suitable for RCS exposure [43].

Importantly, most of the cases of silicosis reviewed were identified through a “passive” surveillance of subjects that were referred to medical attention for transplant evaluation. This fact may impede the diagnosis of many other cases of AS silicosis and prevent an adequate and prompt identification of the public and occupational health impact of “AS silica-related-effects” [7,12]. Although this review fills a gap in the literature, some limitations of our methodology should be carefully considered when drawing conclusions from reported results. Given that knowledge on the topic is still in the early stages, the inclusion criteria range was quite broad, and studies that varied widely in terms of the assessed outcomes and the variables investigated were included to avoid the loss of valuable information. However, the findings reported could not be easily integrated and they were evaluated individually in an attempt to determine common evidence. It is also important to note that all the studies included were observational in nature, and therefore they have inherent biases that should be taken into account when interpreting the results. According to the JBI checklist for case series studies, half for the reviewed papers resulted in good quality, while the others could be classified in fair or poor quality rating [40]. Deficiencies in the methodology and reporting of these latter studies may regard self-selection biases, as they do not clearly detail the inclusion and exclusion criteria for the studied population. Additionally, the use of subjective measures (e.g., self-reporting, unverified information on exposure and preventive measures in the workplace) may also lead to information bias. Furthermore, only a qualitative approach could be used in the review and no quantitative issues relative to the emergence of AS-associated silicosis could be extrapolated. Overall, although all of these issues may question the reliability of the investigations reviewed, the relevance that the topic has for the health, and safety of exposed workers makes it necessary to pursue the most inclusive approach in order to achieve a more substantial understanding of these emerging occupational risks. In this perspective, future longitudinal studies should be planned to make an in-depth assessment of the epidemiological impact that AS working may have on the occurrence of silicosis cases, also in comparison to traditional silica exposure settings. Moreover, these studies should investigate and determine the pathogenesis of the disease in relation to the extent of occupational exposure.

## 5. Conclusions

Over the past few decades, great attention has been paid to the emergence of silicosis cases that are associated with occupational exposure to silica dust generated by the manufacturing, finishing, and installation of AS kitchen and bathroom countertop products, both in fabrication shops and during in-home assembly procedures. Our systematic review enabled us to observe that the clinical characteristics of AS-associated silicosis were comparable to those that were reported for the disease occurring in traditional workplace settings. However, it is important to note that the lack of information concerning silica exposure levels during AS work activities, and the limited awareness regarding silica-derived risks in such innovative applications may have been responsible for the inadequate protection of the workers involved. Further research should aim to fill these gaps in order to better understand AS silicosis pathogenesis, especially in relation to workplace silica concentrations and specific job tasks. It should also investigate and determine the effectiveness of collective and personal protective equipment so as to induce employers, employees, and all factory figures that are engaged in prevention to take concerted action to define/adopt proper measures for protecting the health of exposed workers in AS occupational settings. 

**Figure 2:**
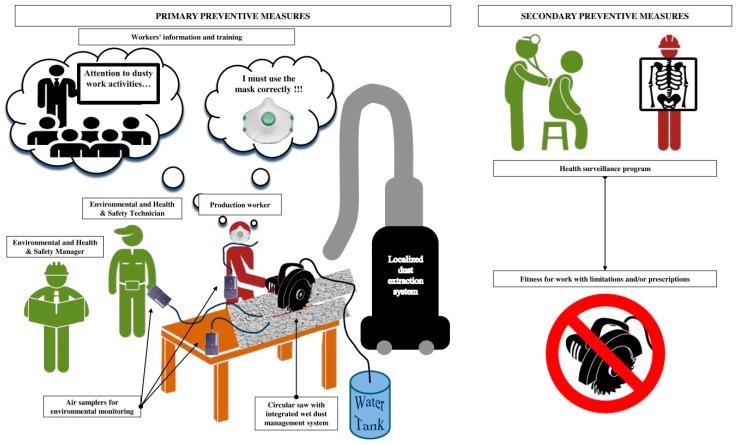
Prevention and protection measures that should be used in artificial stone processing activities.

When considering the critical role of dust exposure control in preventing the development of disease, inadequately controlled RCS concentrations represent a missed opportunity for preventing silicosis that is caused by a known hazardous material. In this respect, exposure control should take the differing composition of the material and the particular work conditions in the AS industry into careful consideration, since these may include high-intensity and short-duration exposures requiring a specifically focused preventive approach. In this scenario, environmental monitoring campaigns should be actively encouraged as a primary preventive measure for assessing levels of exposure to RCS in workplaces during different job tasks and for verifying the efficacy of engineered and personal protective methods of controlling such hazardous exposures (Figure 2). Furthermore, health surveillance is recommended for workers that are exposed to RCS, also in these emerging occupational contexts, in order to achieve the early identification of disease and minimize its potentially severe manifestation (Figure 2). On the other hand, clinicians should be careful when correlating respiratory signs and symptoms of patients with hazardous RCS occupational exposures. 

Outbreaks of silicosis due to AS working can be expected to occur in other countries in the near future if the risks that are associated with the manufacturing or working of engineered stone are not urgently recognized by managers and workers, or well-defined precautionary preventive programs are not suitably applied. A delay in recognizing well-known health hazards in innovative occupational settings may lead to ongoing dangerous exposures and the appearance of further cases. Indeed, in addition to the articles that were reviewed and presented in this review, during the selection process, we identified several other studies (not included in the review, since they did not meet the inclusion criteria, being mostly abstracts, letters to the editor, case-reports, or articles written in languages other than English) that suggest both the presence of AS-associated silicosis in other countries (i.e., the United States, Italy, or Belgium), and a growing interest of the international scientific community in this issue [22,24,25,26,27,28,29,30,31,32,33,34,35,37].

Product stewardship may be helpful in avoiding the mishandling of potentially dangerous materials, and safety datasheets may assist in the identification of the dangerous properties of crystalline silica-containing products. In addition, manufacturers should also be actively involved in communicating the risks of manufacturing/working with hazardous products and in providing resources for the adoption of preventive and protective measures to control harmful exposures [12]. Information and training of the workforce with regard to the possible silicosis risks derived from crystalline silica exposure during the manufacturing and finishing of AS materials, along with suitable health surveillance plans that are designed to recognize cases and case clusters should be actively promoted (Figure 2). Governmental agencies can contribute to prevention, not only by setting and implementing protective exposure standards, but also by giving health and safety support to the companies involved. 

Summing up, stakeholders, manufacturers, occupational risk prevention services, insurance companies for occupational accidents and diseases, business owners, occupational health physicians, general practitioners, and also employees should be engaged, not only in designing/planning processes and operational working environments, but also in assessing the global applicability of proactive preventive and protective measures to identify and control crystalline silica exposure, especially in new and unexpected exposure scenarios, the full extent of which cannot yet be accurately predicted.

## Figures and Tables

**Table 1:** Main characteristics of the artificial stone associated silicosis cases and of the epidemiological studies that investigated this topic.

Country	Study Period	Type of Study	Working Activities Investigated and Correlated Cases of Silicosis (n.)	Age of Workers (Years)	Exposure Time (Years)	Quality Rating by JBI	Reference
Australia	2011–2016	Epidemiological study investigating the prevalence of artificial stone associated silicosis	Dry cutting and polishing of artificial stone for fabrication of small kitchen and bathroom benchtop (7)	44 (median)	7.3 (median)	Fair	Hoy et al. [17]
Israel	1997–2010	Retrospective analysis of patients (with a diagnosis of silicosis) candidates to lung transplantation	Dry cutting of synthetic stone material (Caesar Stone containing ˃85% crystalline silica) for kitchens and other countertop applications (25)	52 (median)	17 ± 9–22 ± 7 (mean ± SD)	Good	Kramer et al. [7]
Israel	1997–2012	Retrospective analysis of patients (with a diagnosis of silicosis) candidates to lung transplantation	Dry cutting and polishing synthetic stone material (with high content of crystalline silica) for kitchens and other countertop applications (40 whom 9 with autoimmune disease)	44.1 (n. 9 -mean); 50.4 (n. 31-mean)	6–26 (9 with autoimmune disease)	Good	Shtraichman et al. [41]
Israel	1997–2015	Evaluation of patients with diagnosis of silicosis visited in a pulmonary outpatient clinic	Dry cutting and polishing artificial decorative stone products (˃93–94% crystalline silica) for kitchens and other countertop applications (82)	47.26 (mean)	19.8 ± 9.4 (mean ± SD)	Fair	Grubstein et al. [42]
Israel	2006–2013	Retrospective analysis of patients who underwent lung transplantation for silicosis	Occupations carrying out job tasks consistent with over-exposure to silica through handling artificial stone (17)	50 (median)	Not reported	Good	Rosengarten et al. [16]
Spain	2008–2011	Prospective observational study investigating the prevalence of silicosis in subjects who worked quartz conglomerates	Cutting, polishing and assembling quartz conglomerates composed of at least 90% natural quartz (crystallized silicon dioxide [SiO_2_] and silica) (6)	39.81 (mean)	12.54 (mean)	Poor	Pascual et al. [44]
Spain	2009–2012	Epidemiological study investigating the prevalence of artificial stone associated silicosis and the correlated working conditions in workers exposed to quartz conglomerates	Working activities (cutting, shaping and finishing) in which agglomerated quartz was used in the manufacturing of countertops for kitchens (46)	33 (median)	12.8 (mean)	Good	Perez-Alonso et al. [43]
Spain	2009–2016	Descriptive epidemiological study assessing the prevalence of artificial stone associated silicosis among the silicosis cases reported to the Healthcare Information System for Occupational Epidemiological Surveillance of the Community of Valencia	Cutting, sanding and assembling artificial quartz aggregates (with a high content of crystalline silica: 70–90%) for kitchen and bath countertops (13)	46.62 ± 13.33 (mean ± SD)	11.00 ± 3.58 (mean ± SD)	Poor	Pascual et al. [45]

JBI, Joanna Briggs Institute Systematic Reviews Checklist for Case Series. Quality rating: good (≥80% positive responses); fair (60–70% positive responses); poor (<60% positive responses); SD, standard deviation.

**Table 2:** Main clinical characteristics of artificial stone associated silicosis reported in the articles included in the review.

Country	Cases (n.)	Respiratory Function Tests	Radiological Assessment	Diagnosis	Reference
Australia	7	3 restrictive defects; 3 mixed obstructive/restrictive defects; 1 normal respiratory function test.	High-resolution computerized tomographic: semiconfluent nodules in the mid and upper zones, ground glass nodules, bilateral upper lobe fibrosis and volume loss with reticulonodular and large confluent mass-like densities	6 with progressive massive fibrosis; 1 chronic silicosis.	Hoy et al. [17]
Israel	25	Moderate to severe restrictive lung disease	Diffuse micronodular pattern and progressive massive fibrosis	2 with progressive massive fibrosis (consistent with accelerated silicosis); 23 chronic silicosis.	Kramer et al. [7]
Israel	9	Restrictive lung disease (8); Normal (1).	Chest X-ray: reticulonodular interstitial pattern (89%); High-resolution computerized tomographic: lymphadenopathy (with or without calcification), alveolar infiltrates, ground glass opacities	Silicosis	Shtraichman et al. [41]
Israel	82	Reduced FEV_1_: 68.4±26 (mean±SD)	High-resolution computerized tomographic: centrilobular and perilymphatic nodules, nodal enlargement with or without nodal calcification, emphysema, and conglomerate masses–progressive massive fibrosis	31 with progressive massive fibrosis (consistent with accelerated silicosis); 51 chronic silicosis.	Grubstein et al. [42]
Israel	17	Reduced FEV_1_ (median: 31; 25^th^-75^th^ percentile range: 27-38) TLC (median: 47; 25^th^-75^th^ percentile range: 41-54)	High-resolution computerized tomographic: picture of interstitial lung disease that was consistent withsilicosis in all cases	Silicosis	Rosengarten et al. [16]
Spain	6	Mild and moderate restrictive ventilatory disorder (2); Moderate obstructive ventilatory disorder (1)	Chest X-ray: radiographic patterns of simple chronic silicosis (83.3%) and progressive massive fibrosis (16.66%)	1 with progressive massive fibrosis; 5 chronic silicosis.	Pascual et al. [44]
Spain	46	Very moderately restrictive pattern (42): FEV_1_=85.9±13, FEV_1_/FVC=79.9±5; In 4 cases was observed a more restrictive spirometric profile: FEV_1_= 74.5±14, FEV_1_/FVC=76.6±9.	Chest X-ray: bilateral diffuse micronodular pattern in 80.4% (37) of the cases; High-resolution computerized tomographic: Micronodules in upper lung zones, diffuse ground-glass pattern (3).	4 with complicated chronic silicosis; 42 simple chronic silicosis.	Perez-Alonso et al. [43]
Spain	13	Spirometric data was obtained in 14 silicosis cases. The results of respiratory function tests refer to the total number of cases (findings of patients exposed to artificial quartz aggregates are not specified): 1 mild restrictive ventilatory dysfunction; 6 had obstructive ventilatory dysfunction (1 very severe, 4 moderate and 1 mild).	High-resolution computerized tomographic data were obtained in 14 silicosis cases. The results refer to the total number of cases (findings of patients exposed to artificial quartz aggregates are not specified): micronodular pattern with hilar and mediastinal adenopathies	3 with progressive massive fibrosis; 10 chronic silicosis.	Pascual et al. [45]

**Table 3:** Protective and preventive measures (collective and individual) reported in the articles included in the review.

Country	Cases (n.)	Environmental Monitoring	Collective Protective Measures	Individual Protective Measures	Reference
Australia	7	Environmental monitoring data not known or available	Poor use of water dust suppression (usually only when polishing activities were performed); Ceiling extraction fans or passive airflow through open doors or windows.	Availability of respiratory protective equipment (disposable masks) was reported only in 3 cases; Lack of information and/or training programs; No Health Surveillance program.	Hoy et al. [17]
Israel	25	Environmental monitoring data not known or available	No dust suppression systems or effective local ventilation	The working activities were performed without any personal respiratory protection	Kramer et al. [7]
Israel	9	Environmental monitoring data not known or available	Not reported	Inadequate respiratory protection (not specified)	Shtraichman et al. [41]
Israel	82	Environmental monitoring data not known or available	Not reported	Not reported	Grubstein et al. [42]
Israel	17	Environmental monitoring carried out by the Israel Ministry of Labor has documented that standard working activities (i.e., dry cutting) with artificial stone cause exposure to levels of silica ˃1 mg/m^3^	No dust suppression systems	The working activities were performed without any personal respiratory protection	Rosengarten et al. [16]
Spain	6	Environmental monitoring data not known or available	Machinery equipped with a waterjet cutting system; Work areas equipped with several dust extraction systems; Natural ventilation.	No specific respiratory protection apparatuses were used (at least until 2009)	Pascual et al. [44]
Spain	46	Environmental monitoring of dust levels was never performed in any workplace	Dust suppression systems (water curtains) present in 32.6% of respondents’ workplace; Ventilation system: 10.9%—dust ventilation system worked properly, 54.3% it was ineffective, in the 34.8% doors and windows, were the only form of ventilation.	Use of personal protective equipment (mask, goggles, helmet, gloves, special footwear and overalls) was referred by 32.6% of the cases. Noteworthy, only three cases reported having constant access to FFP3 or P5 masks; Inadequate periodic preventive medical examinations: only in 8.7% of cases, the health surveillance procedure with execution of chest x-ray was performed periodically.	Perez-Alonso et al. [43]
Spain	13	Environmental monitoring data not known or available	Inadequate ventilation; Presence of dust suppression systems and local ventilation that however are not always used and/or available (i.e., assembly of kitchens and baths is conducted in homes)	Occasional use of individual protection equipment is reported (not specified what type of protective equipment)	Pascual et al. [45]

## References

[1] Leung C.C., Yu I.T., Chen W. (2012). Silicosis. Lancet.

[2] National Institute for Occupational Safety and Health Health Effects of Occupational Exposure to Respirable Crystalline Silica. https://www.cdc.gov/niosh/docs/2002-129/default.html.

[3] Hedlund U., Jonsson H., Eriksson K., Järvholm B. (2008). Exposure–response of silicosis mortality in Swedish iron ore miners. Ann. Occup. Hyg..

[4] Mossman B.T., Churg A. (1998). Mechanisms in the pathogenesis of asbestosis and silicosis. Am. J. Respir. Crit. Care Med..

[5] Lopes-Pacheco M., Bandeira E., Morales M.M. (2016). Cell-Based Therapy for Silicosis. Stem. Cells Int..

[6] Greenberg M.I., Waksman J., Curtis J. (2007). Silicosis: A review. Dis. Mon..

[7] Kramer M.R., Blanc P.D., Fireman E., Amital A., Guber A., Rhahman N.A., Shitrit D. (2012). Artificial stone silicosis [corrected]: Disease resurgence among artificial stone workers. Chest.

[8] Poinen-Rughooputh S., Rughooputh M.S., Guo Y., Rong Y., Chen W. (2016). Occupational exposure to silica dust and risk of lung cancer: An updated meta-analysis of epidemiological studies. BMC Public Health.

[9] Occupational Safety and Health Administration, Department of Labor (2016). Occupational exposure to respirable crystalline silica; final rule. Fed. Regist..

[10] World Health Organization (2007). Elimination of Silicosis GOHNET Newsletter. https://www.who.int/occupational_health/publications/newsletter/gohnet12eref.pdf.

[11] Kauppinen T., Toikkanen J., Pedersen D., Young R., Ahrens W., Boffetta P., Hansen J., Kromhout H., Maqueda Blasco J., Mirabelli D. (2000). Occupational exposure to carcinogens in the European Union. Occup. Environ. Med..

[12] Henneberger P.K., Weissman D.N. (2018). Old adversaries in new places. Occup. Environ. Med..

[13] Maciejewska A. (2008). Occupational exposure assessment for crystalline silica dust: Approach in Poland and worldwide. Int. J. Occup. Med. Environ. Health.

[14] Sato T., Shimosato T., Klinman D.M. (2018). Silicosis and lung cancer: Current perspectives. Lung Cancer.

[15] Steenland K., Ward E. (2014). Silica: A lung carcinogen. CA Cancer J. Clin..

[16] Rosengarten D., Fox B.D., Fireman E., Blanc P.D., Rusanov V., Fruchter O., Raviv Y., Shtraichman O., Saute M., Kramer M.R. (2017). Survival following lung transplantation for artificial stone silicosis relative to idiopathic pulmonary fibrosis. Am. J. Ind. Med..

[17] Hoy R.F., Baird T., Hammerschlag G., Hart D., Johnson A.R., King P., Putt M., Yates D.H. (2018). Artificial stone-associated silicosis: A rapidly emerging occupational lung disease. Occup. Environ. Med..

[18] Ophir N., Shai A.B., Alkalay Y., Israeli S., Korenstein R., Kramer M.R., Fireman E. (2016). Artificial stone dust-induced functional and inflammatory abnormalities in exposed workers monitored quantitatively by biometrics. ERJ Open Res..

[19] Buchanan D., Miller B.G., Soutar C.A. (2003). Quantitative relations between exposure to respirable quartz and risk of silicosis. Occup. Environ. Med..

[20] Cooper J.H., Johnson D.L., Phillips M.L. (2015). Respirable silica dust suppression during artificial stone countertop cutting. Ann. Occup. Hyg..

[21] Meldrum M., Howden P. (2002). Crystalline silica: Variability in fibrogenic potency. Ann. Occup. Hyg..

[22] Guarnieri G., Bizzotto R., Gottardo O., Velo E., Cassaro M., Vio S., Putzu M.G., Rossi F., Zuliani P., Liviero F. (2018). Multiorgan accelerated silicosis misdiagnosed as sarcoidosis in two workers exposed to quartz conglomerate dust. Occup. Environ. Med..

[23] Occupation Safety Health Administration Worker Exposure to Silica during Countertop Manufacturing, Finishing and Installation. https://www.osha.gov/Publications/OSHA3768.pdf.

[24] Bartoli D., Banchi B., di Benedetto F., Farina G.A., Iaia T.E., Poli C., Romanelli M., Scancarello G., Tarchi M. (2012). Silicosis in employees in the processing of kitchen, bar and shop countertops made from quartz resin composite. Provisional results of the environmental and health survey conducted within the territory of USL 11 of Empoli in Tuscany among employees in the processing of quartz resin composite materials and review of the literature. Ital. J. Occup. Environ. Hyg..

[25] Friedman G.K., Harrison R., Bojes H., Worthington K., Filios M. (2015). Centers for Disease Control and Prevention (CDC). Notes from the field: Silicosis in a countertop fabricator—Texas, 2014. MMWR Morb. Mortal Wkly. Rep..

[26] Frankel A., Blake L., Yates D. (2015). Complicated silicosis in an Australian worker from cutting engineered stone countertops: An embarrassing first for Australia. Eur. Resp. J..

[27] García Vadillo C., Gómez J.S., Morillo J.R. (2011). Silicosis in quartz conglomerate workers. Arch. Bronconeumol..

[28] Hoy R. (2017). Artificial stone associated silicosis with rapid lung function decline. Eur. Resp. J..

[29] Jimenez A.L., Hidalgo Molina A., Morales Morales J., Conde Sanchez M.A., Sanchez Romero I., Perez Alonso A., Cordoba Doña J.A. (2017). Rapid clinical progression of silicosis in quartz conglomerate workers in Southern Spain. Eur. Resp. J..

[30] Martínez C., Prieto A., García L., Quero A., González S., Casan P. (2010). Silicosis: A disease with an active present. Arch. Bronconeumol..

[31] Matar E., Frankel A., McCowan Blake L.K., Silverstone E.J., Johnson A.R., Yates D.H. (2017). Complicated silicosis resulting from occupational exposure to engineered stone products. Med. J. Aust..

[32] Paolucci V., Romeo R., Sisinni A.G., Bartoli D., Mazzei M.A., Sartorelli P. (2015). Silicosis in Workers Exposed to Artificial Quartz Conglomerates: Does It Differ From Chronic Simple Silicosis?. Arch. Bronconeumol..

[33] Pérez-Alonso A., Córdoba-Doña J.A., García-Vadillo C. (2015). Silicosis: Relevant differences between marble workers and miners. Arch. Bronconeumol..

[34] Ronsmans S., Decoster L., Keirsbilck S., Verbeken E.K., Nemery B. (2018). Artificial stone-associated silicosis in Belgium. Occup. Environ. Med..

[35] Shtraichman O., Kramer M.R. (2017). Artificial stone silicosis: The Israel epidemic, current view. Harefuah.

[36] Shtraichman O., Blanc P., Ollech J., Fridel L., Fuks L., Fireman E., Shitrit D., Kramer M. (2014). Outbreak of autoimmune disease in a silicosis cluster linked to high-silica content artificial stone. Eur. Res. J..

[37] Barber C.M., Fishwick D., Seed M.J., Carder M., van Tongeren M. (2018). Artificial stone-associated silicosis in the UK. Occup. Environ. Med..

[38] Pavan C., Polimeni M., Tomatis M., Corazzari I., Turci F., Ghigo D., Fubini B. (2016). Editor’s Highlight: Abrasion of Artificial Stones as a New Cause of an Ancient Disease. Physicochemical Features and Cellular Responses. Toxicol. Sci..

[39] Moher D., Liberati A., Tetzlaff J., Altman D.G., PRISMA Group (2010). Preferred reporting items for systematic reviews and meta-analyses: The PRISMA statement. Int. J. Surg..

[40] The Joanna Briggs Institute Critical Appraisal tools for use in JBI Systematic Reviews Checklist for Case Series. http://joannabriggs.org/assets/docs/critical-appraisal-tools/JBI_Critical_Appraisal-Checklist_for_Case_Series2017.pdf.

[41] Shtraichman O., Blanc P.D., Ollech J.E., Fridel L., Fuks L., Fireman E., Kramer M.R. (2015). Outbreak of autoimmune disease in silicosis linked to artificial stone. Occup. Med..

[42] Grubstein A., Osnat Shtraichman M.D., Fireman E., Bachar G.N., Noach-Ophir N., Kramer M.R. (2016). Radiological Evaluation of Artificial Stone Silicosis Outbreak: Emphasizing Findings in Lung Transplant Recipients. J. Comput. Assist. Tomogr..

[43] Pérez-Alonso A., Córdoba-Doña J.A., Millares-Lorenzo J.L., Figueroa-Murillo E., García-Vadillo C., Romero-Morillos J. (2014). Outbreak of silicosis in Spanish quartz conglomerate workers. Int. J. Occup. Environ. Health.

[44] Pascual S., Urrutia I., Ballaz A., Arrizubieta I., Altube L., Salinas C. (2011). Prevalence of silicosis in a marble factory after exposure to quartz conglomerates. Arch. Bronconeumol..

[45] Pascual Del Pobil Y., Ferré M.A., García Sevila R., García Rodenas M.D.M., Barroso Medel E., Flores Reos E., Gil Carbonell J. (2019). Silicosis: A former occupational disease with new occupational exposure scenarios. Rev. Clin. Esp..

[46] Rosenman K.D., Reilly M.J., Gardiner J. (2010). Results of spirometry among individuals in a silicosis registry. J. Occup. Environ. Med..

[47] Di Giuseppe M., Gambelli F., Hoyle G.W., Lungarella G., Studer S.M., Richards T., Yousem S., McCurry K., Dauber J., Kaminski N. (2009). Systemic inhibition of nf-kappab activation protects from silicosis. PLoS ONE.

[48] Enfield K.B., Floyd S., Barker B., Weder M., Kozower B.D., Jones D.R., Lau C.L. (2012). Survival after lung transplant for coal workers’ pneumoconiosis. J. Heart Lung Transplant..

[49] Directive (EU) 2017/2398 Of The European Parliament And Of The Council of 12 December 2017 Amending Directive 2004/37/EC on the Protection of Workers from the Risks Related to Exposure to Carcinogens or Mutagens at Work. https://eur-lex.europa.eu/legal-content/EN/TXT/PDF/?uri=CELEX:32017L2398&from=EN.

[50] Phillips M.L., Johnson D.L., Johnson A.C. (2013). Determinants of respirable silica exposure in stone countertop fabrication: A preliminary study. J. Occup. Environ. Hyg..

[51] Johnson D.L., Phillips M.L., Qi C., Van A.T., Hawley D.A. (2017). Experimental Evaluation of Respirable Dust and Crystalline Silica Controls During Simulated Performance of Stone Countertop Fabrication Tasks With Powered Hand Tools. Ann. Work Expo. Health.

